# Gallstone Ileus After Conservative Management of Acute Cholecystitis and Refusal of Interval Cholecystectomy: A Case Report

**DOI:** 10.3390/reports9030234

**Published:** 2026-07-22

**Authors:** Hussain Alessa, Afnan Alshayeb, Renad Aljasser, Abdulaziz Ali Qahtani

**Affiliations:** 1 Department of General Surgery, Ministry of the National Guard Health Affairs, AlMubarraz 39182, Saudi Arabia; 2 Medical College, Dar Al Uloom University, Riyadh 13314, Saudi Arabia

**Keywords:** gallstone ileus, acute cholecystitis, small bowel obstruction, cholecystoenteric fistula, enterolithotomy

## Abstract

**Background and Clinical Significance**: Gallstone ileus (GI) is a rare but serious complication of gallstone disease. This condition is characterized by migration of gallstones through a cholecystoenteric fistula, resulting in mechanical bowel obstruction. GI primarily affects older patients with multiple comorbidities. **Case Presentation**: Herein, we describe a case of 68-year-old Saudi woman with a history of end-stage renal disease, pulmonary hypertension, diabetes mellitus, bronchial asthma, and atrial fibrillation. She presented with acute calculous cholecystitis. Due to her condition, she was managed conservatively initially. The patient was recommended laparoscopic cholecystectomy but she declined due to fear of anesthesia-related complications. She re-presented after two months with abdominal pain, vomiting, distension, and constipation. Computed tomography (CT) scan showed a 3.2 cm ectopic gallstone impacted in the distal ileum with proximal bowel dilatation and segmental ischemia, confirming GI. Laparotomy showed bowel ischemia and localized perforation. The patient was managed with enterolithotomy, resection of 25 cm of distal ileum, and primary anastomosis. Post-surgery, the patient had favorable recovery. **Conclusions**: This case highlights need for early diagnosis and appropriate management of GI in patients with multiple comorbidities.

## 1. Introduction and Clinical Significance

Gallstone ileus (GI) is a rare but life-threatening mechanical intestinal obstruction caused by passage of gallstone through a bilioenteric fistula. Although gallstone disease is highly prevalent worldwide, GI develops in only 0.3–0.5% of patients with cholelithiasis and accounts for approximately 1–4% of all cases of small bowel obstruction (SBO) [1,2]. GI primarily affects older females with chronic calculous cholecystitis [3,4]. Mortality ranges from 12% to 27%. The high mortality can be attributed to delayed diagnosis, advanced age, and multiple comorbidities [3,5]. The literature shows a median delay of 2–37 days between admission and surgical intervention [6]. This delay can contribute to intestinal ischemia and sepsis. GI can be misdiagnosed because its clinical manifestations like nausea, vomiting, colicky pain and constipation overlap with other causes of SBO. Radiologically, Rigler’s triad consisting of pneumobilia, ectopic gallstone, and intestinal obstruction remains the classical diagnostic finding but it is identified in only 15–50% of patients [5,7,8]. Management remains controversial. Conservative management of acute cholecystitis in high-risk individuals can defer immediate complications but increases the probability of later GI, biliary sepsis, or perforation [5,9].

This case illustrates these dilemmas through a 68-year-old woman who developed GI following refusal of elective cholecystectomy after conservative treatment for acute cholecystitis. It underscores the clinical risks of nonoperative management in multimorbid patients and highlights the diagnostic and therapeutic challenges faced in regions such as Saudi Arabia where the literature on GI remains limited. This case report has been reported in line with the SCARE criteria [10].

## 2. Case Presentation

A 68-year-old Saudi woman with type 2 diabetes mellitus, hypertension, end-stage renal disease (ESRD) on thrice-weekly hemodialysis, bronchial asthma, pulmonary hypertension requiring nocturnal BiPAP, and paroxysmal atrial fibrillation on apixaban (2.5 mg twice daily) presented with fever, vomiting, and upper abdominal pain. Pain began one week earlier, dull and radiating to the back, with an 8 kg unintentional weight loss over two months. She denied jaundice, hematemesis, or melena.

**Figure 1:**
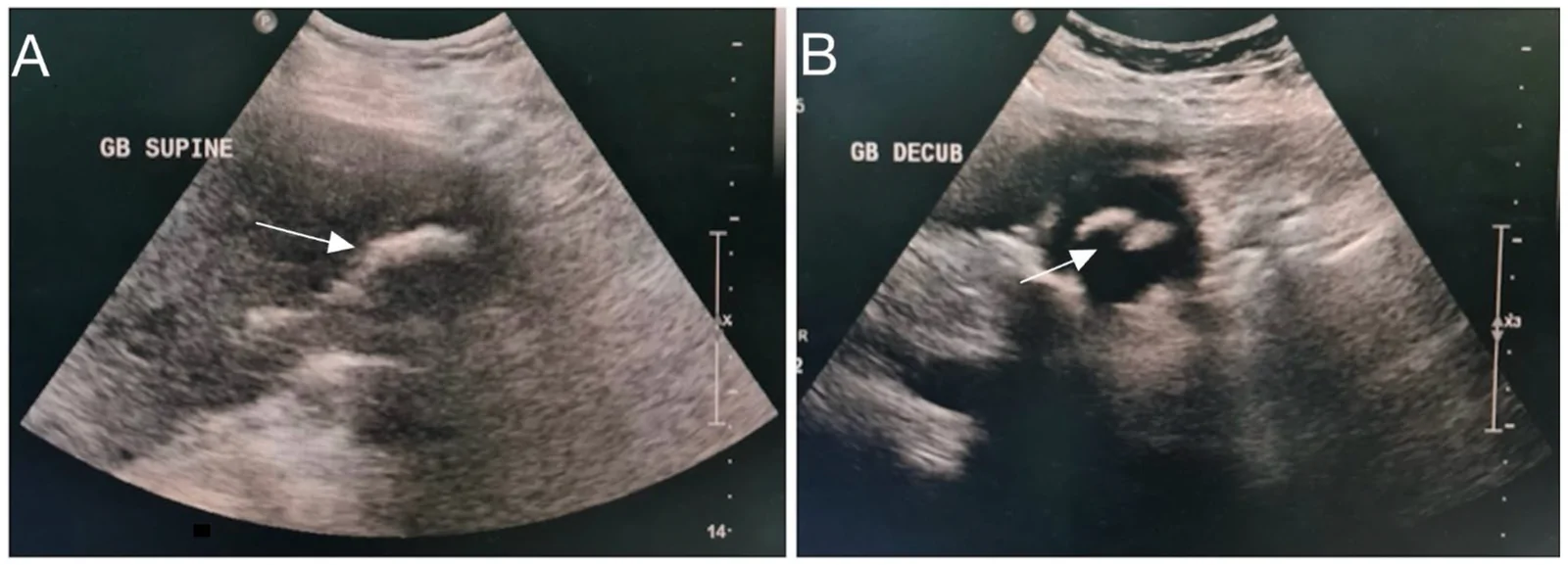
Ultrasonography detected gallstones (arrow). (**A**) Supine position showing the presence of a gallstone, (**B**) decubitus position confirming the same stone with a different angle of view.

On initial evaluation, the patient appeared clinically unwell and hypotensive, with a blood pressure of 75/44 mmHg, heart rate of 73 beats/min, respiratory rate of 20 breaths/min, oxygen saturation of 96% on room air, and no documented fever at the time of examination. Abdominal examination revealed mild epigastric tenderness without guarding or rigidity. Laboratory tests showed leukocytosis (WBC 33 × 10^9^/L), AST 55 U/L, ALT 35 U/L, and markedly elevated troponin I (4256 ng/L). Electrolytes reflected mild metabolic acidosis secondary to renal disease. Chest radiography showed pacemaker leads and bilateral perihilar congestion. Ultrasound revealed gallstones without wall thickening or pericholecystic collection (Figure 1).

**Figure 2:**
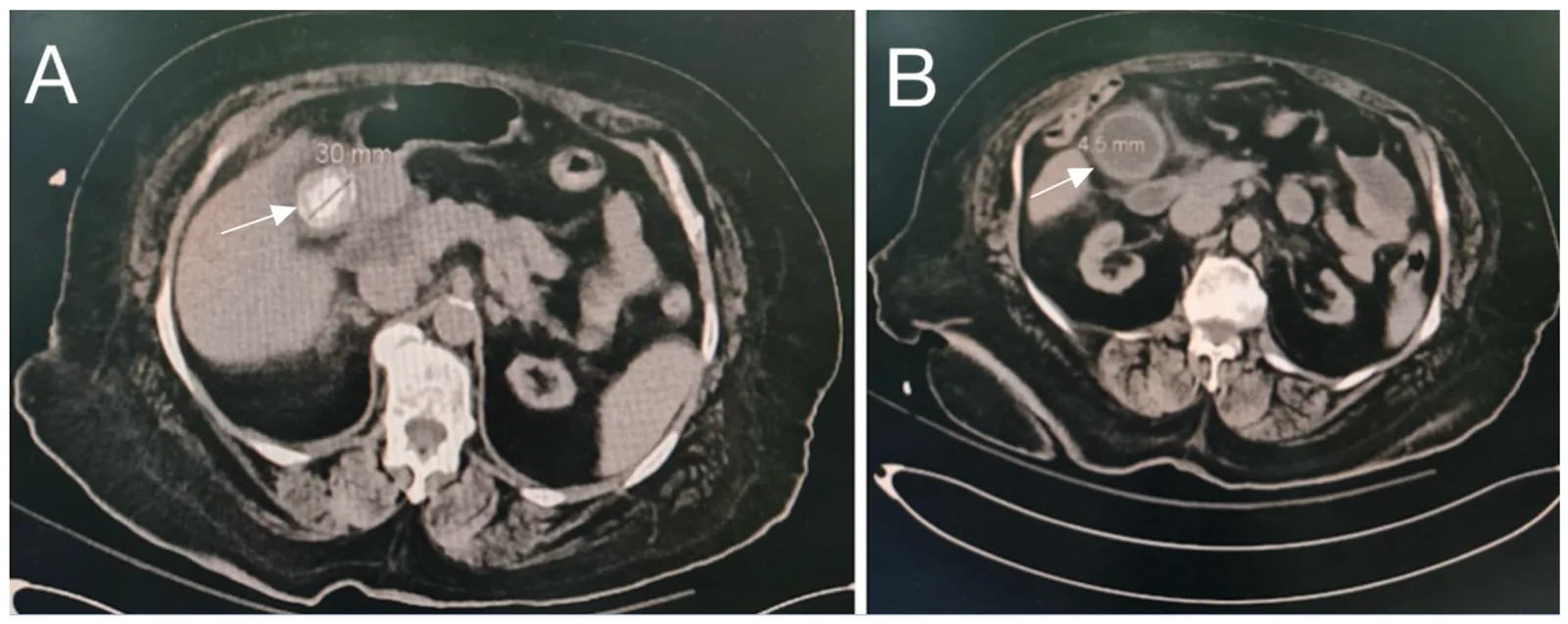
CT scan of the abdomen and pelvis: (**A**) the gallbladder demonstrates an intraluminal hyper-dense stone located in the fundus, measuring 3 cm (arrow). (**B**) thickening of the gallbladder wall (0.4 cm) with surrounding fat stranding and minimal pericholecystic free fluid, indicative of inflammation (arrow).

Contrast-enhanced CT demonstrated a 3 cm gallstone with gallbladder wall thickening and pericholecystic fluid, confirming acute cholecystitis (Figure 2).

Several differential diagnoses were considered at presentation, including acute pancreatitis, perforated peptic ulcer disease, mesenteric ischemia, and myocardial ischemia. Pancreatitis was excluded by normal serum amylase and lipase. Absence of pneumoperitoneum excluded perforation, while CT localized inflammation to the gallbladder. Cardiology consultation attributed the markedly elevated troponin level to demand ischemia secondary to sepsis and hemodynamic instability rather than acute coronary syndrome. Given her prohibitive operative risk from renal, cardiac, and pulmonary comorbidities, she was treated conservatively with IV piperacillin–tazobactam, fluids, and careful dialysis scheduling. The patient’s condition improved with conservative management, and she was discharged after a six-day hospitalization. At discharge, interval laparoscopic cholecystectomy was recommended once she was medically optimized; however, after detailed counseling regarding the risks and benefits, the patient declined the procedure because of concerns about anesthesia-related complications. During the initial admission, no treatment-related adverse effects, including antibiotic-associated complications or procedure-related events, were observed.

**Figure 3:**
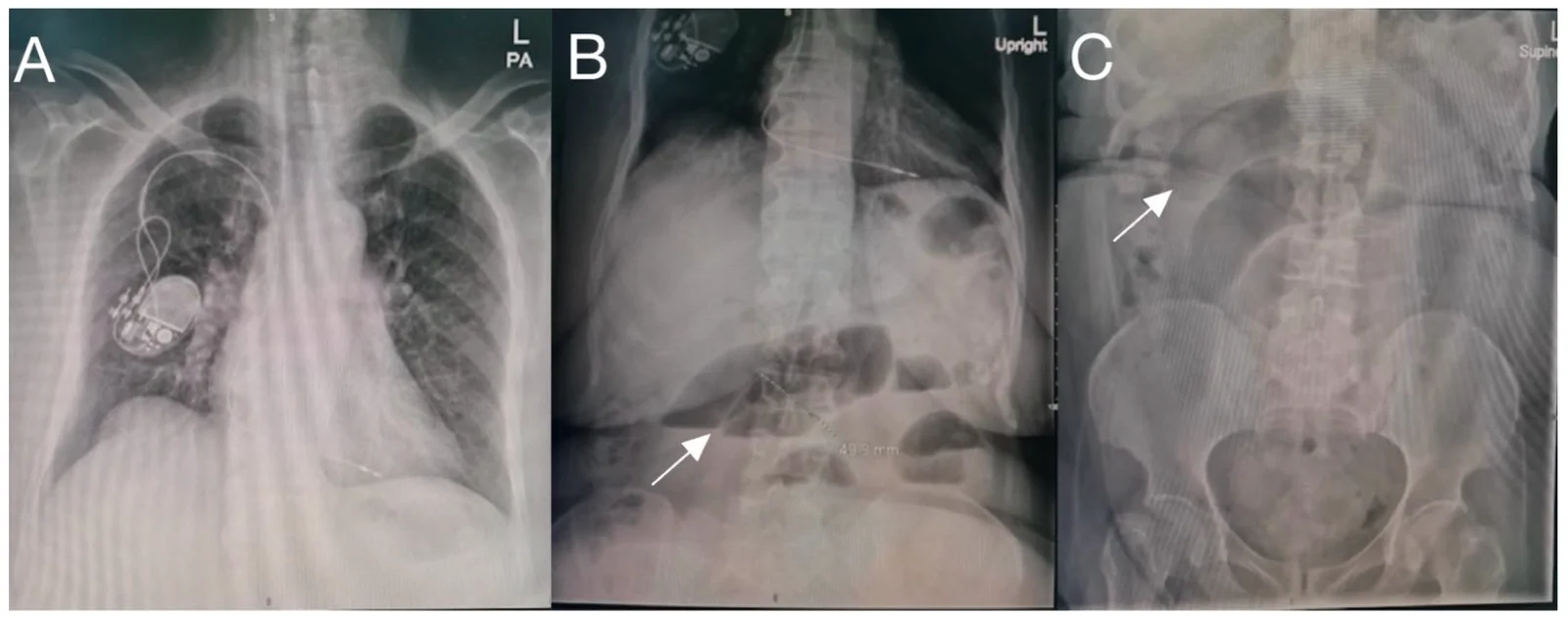
Plain X-ray: (**A**) PA chest X-ray showing an unremarkable study with no evidence of air under the diaphragm. (**B**,**C**) Abdominal X-ray showing prominent small bowel loops (arrow), suggestive of a potential obstruction.

Two months later, the patient re-presented with severe epigastric abdominal pain, persistent nausea and vomiting, progressive abdominal distension, and absolute constipation for ten days. Physical examination demonstrated abdominal distension with high-pitched bowel sounds, raising concern for mechanical small bowel obstruction. The patient was hemodynamically stable with a heart rate of 60 beats/min, blood pressure of 126/53 mmHg, oxygen saturation of 100% on room air, and a normal respiratory rate. Laboratory investigations demonstrated white blood cell count of 11.5 × 10^9^/L, serum creatinine of 701 µmol/L, ALT of 16 U/L, AST of 23 U/L, and D-dimer of 1.5 mg/L. Plain abdominal radiographs revealed multiple dilated bowel loops with air-fluid levels suggestive of intestinal obstruction (Figure 3).

**Figure 4:**
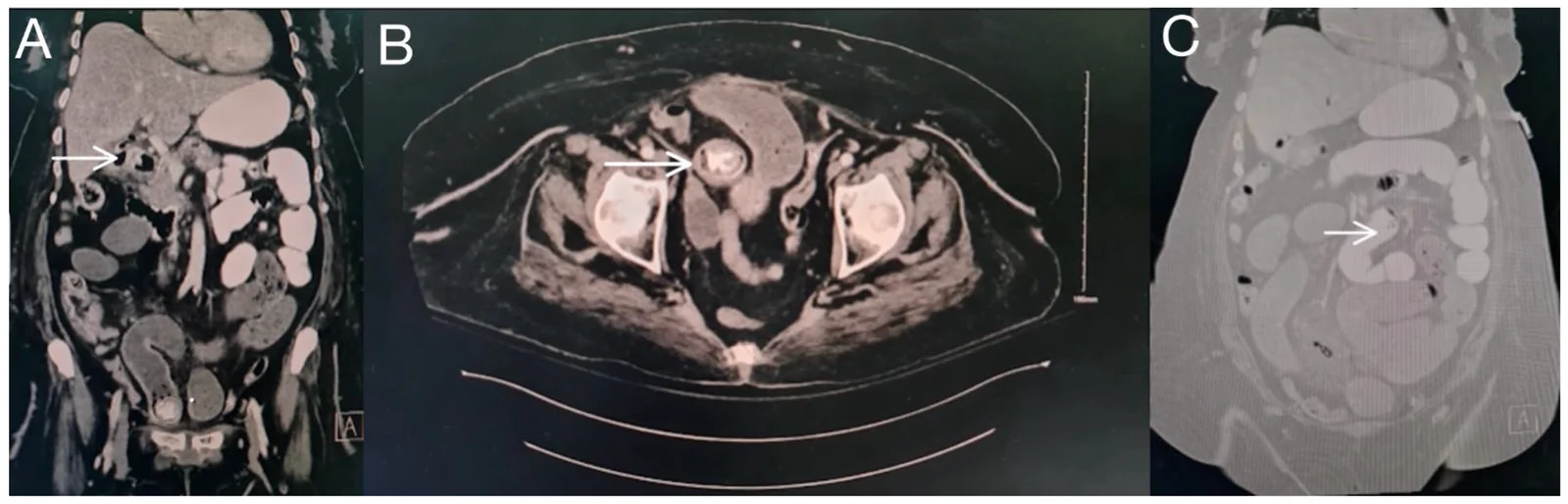
CT abdomen and pelvis: (**A**) coronal view showing the previously noted sizable gallstone not visualized in the gallbladder, with the gallbladder appearing contracted. Positive oral contrast is seen filling the gallbladder and the common bile duct (CBD); findings were suggestive of a bilioenteric fistula, most likely cholecystoduodenal, although the fistula was not surgically explored (arrow). Minimal pneumobilia is also noted. (**B**) Axial view showing the previously noted sizable gallstone (3.2 × 2.6 cm) now located in the distal ileum (arrow). (**C**) Coronal view demonstrating foci of extra-luminal air (arrow), suggestive of small bowel wall pressure necrosis or ischemia.

Abdominal ultrasonography was limited by excessive bowel gas and did not provide additional diagnostic information. Repeat contrast-enhanced CT imaging demonstrated a 3.2 cm ectopic gallstone impacted within the distal ileum, associated with proximal small bowel dilatation, mesenteric fat stranding, and radiological evidence of segmental bowel ischemia (Figure 4), confirming GI.

**Figure 5:**
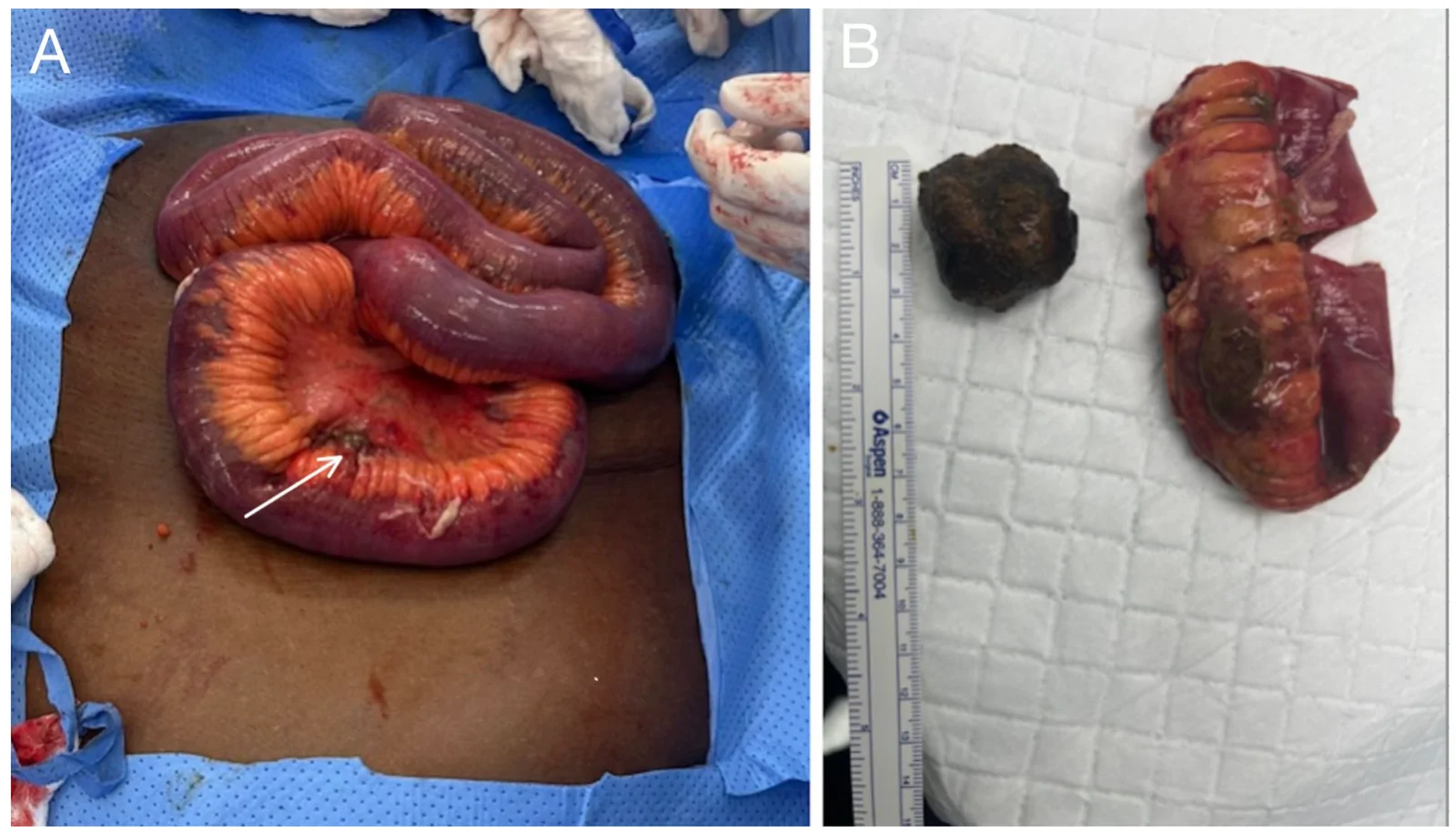
(**A**) Exploratory laparotomy revealed a large gallstone impacted in the ileum with surrounding ischemia and localized perforation (arrow). (**B**) Enterolithotomy with segmental small bowel resection was performed.

The patient underwent urgent exploratory laparotomy. Intraoperatively, a large gallstone was identified approximately 60 cm proximal to the ileocecal junction. The involved bowel segment demonstrated ischemic changes with localized perforation. Enterolithotomy with resection of approximately 25 cm of distal ileum and primary anastomosis was therefore performed (Figure 5).

Preoperative coagulation studies showed hemoglobin 12 g/dL, platelet count 273 × 10^9^/L, PT 14.6 s, INR 1.4, aPTT 33.6 s, and fibrinogen 5.6 g/L. Because the patient had received apixaban within 24 h before surgery, perioperative bleeding risk was substantial and required multidisciplinary management with transfusion support, including ten units of cryoprecipitate, six units of platelets, four units of packed red blood cells, and four units of fresh frozen plasma.

Postoperatively, the patient was transferred to the intensive care unit for close monitoring. She required short-term mechanical ventilation and was successfully extubated on postoperative day one. Her postoperative recovery was otherwise uneventful, with gradual return of bowel function and stabilization of laboratory parameters. She was discharged home on postoperative day ten in stable condition. Timeline for clinical events in this case is presented in Table 1.

## 3. Discussion

Although several cases of GI have been published, there is still a paucity of case reports from Saudi Arabia. There are several unique aspects of this case. For instance, GI developed after refusal of cholecystectomy despite prior counseling which led to disease progression. The patient also had history of ESRD. Chronic renal failure on dialysis compounds GI risk. CKD patients have very high gallstone prevalence up to 46.9% in stage 5 CKD [11]. Uremia also predisposes to bleeding diathesis and poor healing. Furthermore, dialysis alone only minimally clears apixaban. Mavrakanas et al. reported that even low-dose apixaban (2.5 mg twice daily) in ESRD leads to drug exposure comparable to a full dose in normal kidneys [12]. Many GI cases are treated with enterolithotomy alone if the bowel appears viable [7]. In our patient, the impacted stone had caused segmental ileal ischemia and a contained perforation which is an unusually severe complication. This necessitated resection of 25 cm of distal ileum with primary anastomosis. Bowel resection increases operative time and risk, especially in the setting of recent apixaban exposure and increased perioperative bleeding risk. This highlights the acuity of the situation. Such advanced GI with perforation is rare and has high morbidity [13]. Eiref et al. reported a case of a 67-year-old female who reported perforation of multiple segments of small bowel (mid-ileum and mid-jejunum) caused by GI [14]. To our knowledge, reports from Saudi Arabia specifically describing gallstone ileus following patient refusal of interval cholecystectomy remain limited.

GI remains rare but contributes disproportionately to morbidity in elderly patients. The mean age at diagnosis is 65–75 years, and 70–80% of cases occur in women [3,4,7]. GI occurs when chronic cholecystitis leads to an erosion between the gallbladder and adjacent gut which allows large gallstones to migrate into the bowel. The distal ileum is the most common site of impaction (60.5%), followed by jejunum (16.1%), stomach (14.2%), colon (4.1%), and duodenum (3.5%) [15,16]. The ileum is most frequently affected due to its narrow lumen and slower transit. Clinical presentation of GI may mimic SBO [17]. Our patient’s initial re-presentation with high-pitched bowel sounds and radiographs with air-fluid levels were consistent with SBO. CT abdomen is far more sensitive and specific and identifies intraluminal gallstones, pneumobilia, and obstruction sites in the majority of cases [18]. In this case, CT promptly revealed a 3.2 cm stone impacted in the distal ileum, bowel dilation, and focal ischemic changes, confirming GI. CT has a sensitivity and specificity of 93% and 100%, respectively [18].

Up to 30% present with concurrent acute cholecystitis, but fewer than 15% show jaundice [19]. The intermittent nature of obstruction (“tumbling phenomenon”) leads to variable symptoms and diagnostic delay averaging 6–8 days [20]. Surgery is the mainstay of GI management but choice of procedure is individualized by patient condition and intraoperative findings. Enterolithotomy alone achieves immediate relief with mortality rates around 11.7%, compared with 16.9% for one-stage repair [21]. Although enterolithotomy carries a 5–17% risk of recurrent biliary events, the physiologic stress of definitive repair often outweighs its benefit in elderly or comorbid patients [4,22]. The two-stage approach initial enterolithotomy followed by delayed cholecystectomy and fistula closure once stable has become the preferred method in such populations [21]. A one-stage approach combining enterolithotomy, cholecystectomy, and fistula closure reduces recurrence but carries high perioperative mortality in frail patients (12–33%) [8,9]. Isolated enterolithotomy has lower operative risk (5–12% mortality) but up to 17% recurrence due to persistent fistula or residual stones [9].

In our patient, emergent one-stage repair was deemed too risky. She presented with hemodynamic instability and massive comorbid burden, and indeed intraoperatively an enterolithotomy with bowel resection was performed without simultaneous cholecystectomy. This decision aligns with the literature recommending minimal intervention in unstable, highly comorbid patients [17,18]. Our patient’s impending perforation necessitated immediate resection. Her preoperative anticoagulation and dialysis dependence elevated hemorrhagic and metabolic risk. Blood product preparation and perioperative dialysis coordination minimized complications, exemplifying best-practice multidisciplinary care. Recurrent cholecystitis causes chronic inflammation, adhesions, and fistula formation most often between the gallbladder and duodenum (60%), followed by colon (17%) and stomach (5%). Large stones (>2.5 cm) pass through the fistula, migrating distally until impaction [23]. In our case, the 3.2 cm stone obstructed the distal ileum, triggering ischemia from pressure necrosis and bacterial translocation.

This case also highlights the need for shared decision making. The patient’s earlier refusal of elective cholecystectomy illustrates how personal risk perception may diverge from clinical advice. In frail individuals, shared decision-making should explicitly document potential consequences of nonoperative management fistula formation, obstruction, and sepsis.

## 4. Conclusions

GI remains a rare but serious late complication of untreated cholecystitis, particularly among elderly patients with multiple comorbidities. Conservative management may temporarily stabilize symptoms but carries a substantial risk of delayed biliary-enteric fistula and intestinal obstruction. Early recognition through CT imaging, prompt surgical relief of obstruction, and tailored perioperative management are essential to improve outcomes. This case illustrates the potential risk of delayed definitive management following conservative treatment of acute cholecystitis in high-risk patients and emphasizes the importance of early surgical decision-making, multidisciplinary coordination, and regional data collection to enhance clinical preparedness for this uncommon yet life-threatening condition.

## Figures and Tables

**Table 1:** Timeline of clinical events.

Time	Clinical Event
Initial presentation	Fever, vomiting and upper abdominal pain
Day 1	CT confirmed acute calculous cholecystitis
Hospital course	Conservative treatment with IV antibiotics and hemodialysis optimization
Before discharge	Elective laparoscopic cholecystectomy recommended but declined by the patient
Two months later	Presented with abdominal pain, vomiting, distension and constipation
Emergency admission	CT confirmed gallstone ileus with distal ileal obstruction and bowel ischemia
Same day	Emergency laparotomy, enterolithotomy, bowel resection and primary anastomosis
POD 1	Extubated successfully
POD 10	Discharged home in stable condition

POD: postoperative day.

## Data Availability

The original contributions presented in this study are included in the article. Further inquiries can be directed to the corresponding author.
